# Lead levels of new solvent-based household paints in Zimbabwe and Botswana: A preliminary study

**DOI:** 10.4102/phcfm.v14i1.3486

**Published:** 2022-08-30

**Authors:** Rose A. Kambarami, Lucia L. Coulter, Louisa Chikara Mudawarima, Gwen Kandawasvika, Jack Rafferty, Clare Donaldson, Benjamin Stewart

**Affiliations:** 1 Child and Adolescent Health Unit, Faculty of Medicine and Health Sciences, University of Zimbabwe, Harare, Zimbabwe; 2 Lead Exposure Elimination Project, London, United Kingdom

**Keywords:** lead exposure, lead paint, lead poisoning, lead paint elimination, environmental health, toxic metals

## Abstract

**Background:**

Lead paint remains a major potential source of lead poisoning globally, but there has been no documentation on lead content in solvent paints available on the markets in Zimbabwe and Botswana.

**Aim:**

To determine the lead content of solvent-based paints available on the market in Zimbabwe and Botswana and identify a need for a larger study to inform policy.

**Methods:**

This pilot study was conducted in Harare, Zimbabwe, and Gaborone, Botswana. Popular brands of solvent-based household paints were bought from hardware shops in Harare (10 samples) and Gaborone (19 samples). Samples were analysed for lead content using inductively coupled plasma-atomic emission spectrometry.

**Results:**

Seventy percent of samples from Zimbabwe were found to contain lead above 90 parts per million (ppm), the recommended regulatory limit, with ranges from less than 60 ppm to 12 000 ppm. Twenty percent of Zimbabwean samples had lead levels above 10 000 ppm. No samples from Botswana had lead concentration above the detection limit, with all levels below 100 ppm.

**Lesson Learnt:**

Data strongly suggest very high lead content in popular brands of solvent paints in Zimbabwe, indicating a need for a larger, well-designed study for policy direction.

## Background

Lead is a naturally occurring toxic metal found in the Earth’s crust whose widespread use has resulted in extensive environmental contamination, thus making it a public health threat.^1,2,3^ The various uses of lead include use in petrol, paint, playgrounds, toys, households, glass, cellular phones, computers, protective clothing, candles and cosmetics.^4,5,6^ There is no level of exposure known to be without harm in both adults and children, but children are particularly vulnerable and can absorb four to five times as much ingested lead as adults.^1^ The history of lead distribution in the human environment and its recognition as a neurotoxin spans over 2000 years,^5^ and yet there are countries with no regulations for lead in paint.^7^ Lead is neurotoxic and even low levels of exposure in childhood can result in reduced intelligence, increased intellectual disability rates, lower educational attainment and reduced future earnings.^8,9,10,11,12^ Lead affects all body systems and can cause anaemia, growth stunting and renal and cardiovascular diseases later in life.^8,11,12,13,14^

One of the most well-evidenced and feasible sources of childhood lead poisoning to address is lead paint.^15^ Lead paint is a primary source of lead poisoning globally and is widespread in Africa.^3,6,11,16,17^ There has been no research to date investigating whether paint is a source of lead exposure in Zimbabwe. Zimbabwe currently has no lead paint.^7^

### Objective

To conduct a preliminary study to determine whether there is lead in solvent-based paint for home use currently being sold in Zimbabwe and Botswana and provide evidence for a larger study to further characterise the problem and inform the development of lead paint regulation.

## Methods

### Sample collection and preparation

Common brands of solvent-based paint were purchased from popular hardware stores in Harare, Zimbabwe, in July 2021. Five major hardware and paint stores were visited to identify all five common brands available; 10 cans of solvent-based paint from these five brands were included. Similarly, 19 cans of solvent-based paint were purchased from stores in Gaborone, Botswana. Fifteen hardware and paint stores were visited, and all seven available brands were included. Between one and three cans of different colours were purchased from each brand, depending on the availability. Information on the paint cans was carefully recorded; then, samples were prepared according to standard methods used elsewhere.^16^ The paint was thoroughly stirred and applied to a polypropylene surface 10 cm by 10 cm across in a 0.5 mm – 1.0 mm coating. Stirring and application was performed with a separate wooden implement for each paint. Samples were allowed to dry before being packaged in clean polyethylene bags and posted to the lab for analysis.

### Sample analysis

The paint from both countries was analysed for lead content based on dry weight by the accredited Wisconsin Occupational Health Laboratory, United States of America, using the National Institute for Occupational Safety and Health 7303 method.^13,18^ The laboratory is accredited by the American Industrial Hygiene Association under the U.S. Environmental Protection Agency’s Environmental Lead Laboratory Accreditation Programme and participates in its Environmental Lead Proficiency Analytical Testing Programme. Paint scrapings were digested using hydrochloric acid, nitric acid and a hot block.^18^ The samples were analysed using inductively coupled plasma-atomic emission spectrometry.

Samples with concentrations below the reporting limit of the analytical procedures used were reported as ‘less than’ (<) the reporting limit. The reporting limit used for the Zimbabwe analysis was 60 parts per million (ppm), and for the Botswana results it varied between 86 ppm and 100 ppm.

### Ethical considerations

The study does not include any interaction or intervention with human or animal subjects. The National Health Research and Development Committee (NHRDC) reviewed and discussed the submitted abstract and advised that it does not meet the Medical Research Council of Zimbabwe criteria for ethical review. The study was granted an exemption from ethical review.

## Results

Table 1 summarises the lead concentration data for the paint samples in Zimbabwe and Botswana. Seventy percent of paint samples from Zimbabwe were above 90 ppm, presenting significant health risks and were above the limit suggested by the World Health Organization (WHO) and United Nations (UN).^15,19^ Sixty percent of samples were above the higher limit of 600 ppm, and 20% of samples were above 10 000 ppm, indicating an extremely high lead concentration. Regarding the samples from Zimbabwe, the mean concentration was 4863 ppm, the median concentration was 6900 ppm and the maximum concentration was at 12 000 ppm.

**TABLE 1:** Summary of lead concentrations in solvent-based paint samples in Zimbabwe and Botswana.

Country	Number of samples	Average of all paints (ppm)	Average of coloured paints (ppm)	% ≥ 90 ppm	% ≥ 600 ppm	% ≥ 10 000 ppm	Minimum (ppm)	Maximum (ppm)
Zimbabwe	10	4863	6919	70	60	20	< 60	12 000
Botswana	19	< 100	N/A	0	0	0	< 86	< 100

ppm, parts per million.

All the 19 paint samples from Botswana were found to be below the laboratory reporting limit. Of the seven brands tested, six were manufactured in South Africa, which had a 600 ppm lead paint law in place.^7^

**FIGURE 1:**
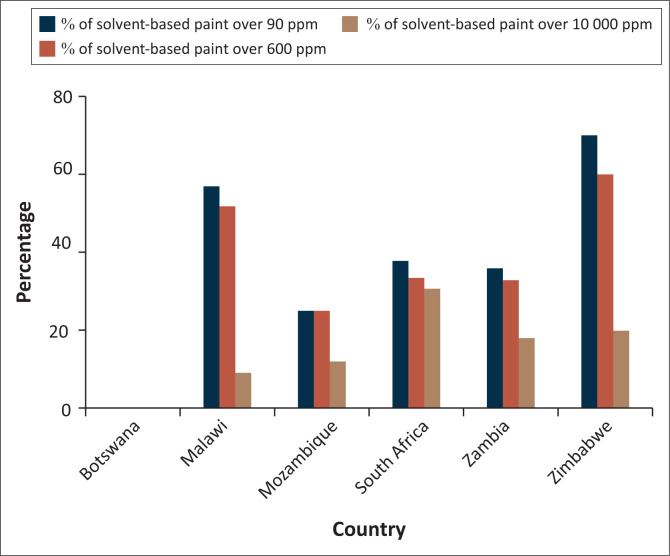
Compares the lead paint exceedance rates at 90 ppm, 600 ppm and 10 000 ppm in this study and previous studies in Southern Africa.^17^

Figure 1 compares the results of this study to previous studies in Southern Africa.^17^ Zimbabwe and Botswana had the highest and lowest rates of lead for the group, respectively. Botswana had the lowest ‘exceedance’ rates (the proportion of samples above a given limit) for the group at 90 ppm, 600 ppm and 10 000 ppm limits. Zimbabwe had the highest exceedance rates for the group at 90 ppm and 600 ppm limits and the second-highest exceedance rate at the 10 000 ppm limit.

The lead content of samples from Zimbabwe was greater in coloured paints. All white or cream paint samples (*n* = 2) did not have lead content above 90 ppm. All (100%) of red, yellow or orange (*n* = 6) samples were above 90 ppm. Fifty percent of green samples (*n* = 2) were above 90 ppm.

Three samples made claims of no added lead on can labels, but two of these had lead concentrations of 6900 ppm. Of the seven samples with no claims about lead content on labels, five had lead concentrations over 90 ppm.

## Discussion

This study found high levels of lead in a majority of paints and brands sampled in Zimbabwe. The average and median concentration of all paints tested was over 50 times the recommended regulatory limit of 90 ppm and sufficient to present serious health risks.^15^ Some tested paints were above 10 000 ppm, an extremely high level that presents even greater health risks.

However, the study also found that 30% of Zimbabwean samples had lead concentrations below 90 ppm. These samples included both white and coloured paints and were manufactured in Zimbabwe. These results suggest that producing and selling lead-free paint is possible in Zimbabwe.

These results support previous studies, which have found that white paint is less likely to contain lead.^16^ This finding suggests that lead is being added to paint in Zimbabwe as a pigment.^3^ Transitioning to non-lead pigments is both feasible and cost-effective.^15^

False claims of ‘no added lead’ were identified in this study. Mandating clear warnings and verified lead-free claims will be an important part of any future regulatory solution.

Paints available in Botswana do not appear to contain high concentrations of lead. However, lead paint may still be present on existing structures. Botswana does not regulate lead paint, so there are no barriers to lead paint entering the market through local producers or importation. Botswana’s positive results are likely because six out of the seven brands were imported from South Africa, which does regulate lead paint. This is a fragile solution, and a more robust remedy would be for Botswana to introduce lead paint regulation.

Regulating the presence of lead in household paints is a clear and effective path to reduce lead exposure.^3^ Regulation that limits lead concentrations in paint to below 90 ppm is a global standard and already in place in 79 countries.^7^

This study was limited by its small sample size, only including 10 paints in Zimbabwe and 19 paints in Botswana. Nevertheless, given that all main brands available in the largest city of each country were represented, it is likely that larger studies of the paint market would corroborate our key finding of excessive lead in Zimbabwe’s paints. Our study is also limited by its focus on paint currently available on the market. Field studies of the lead content of paint on existing surfaces in Zimbabwe and Botswana would further inform the extent to which lead paint is a source of exposure in the two countries.

## Conclusion

This study demonstrates for the first time that some solvent-based paints sold in Zimbabwe contain extremely high levels of lead, suggesting that paint may contribute to lead exposure in the Zimbabwean population. A larger study may provide a clearer picture of the size of the problem, the effects thereof and the need for regulation.
